# Stretchable and anti-impact iontronic pressure sensor with an ultrabroad linear range for biophysical monitoring and deep learning-aided knee rehabilitation

**DOI:** 10.1038/s41378-021-00318-2

**Published:** 2021-11-17

**Authors:** Hongcheng Xu, Libo Gao, Haitao Zhao, Hanlin Huang, Yuejiao Wang, Gang Chen, Yuxin Qin, Ningjuan Zhao, Dandan Xu, Ling Duan, Xuan Li, Siyu Li, Zhongbao Luo, Weidong Wang, Yang Lu

**Affiliations:** 1 School of Mechano-Electronic Engineering, Xidian University, Xi’an, 710071 China; 2 CityU-Xidian Joint Laboratory of Micro/Nano-Manufacturing, Shenzhen, 518057 China; 3 Materials Interfaces Center, Shenzhen Institutes of Advanced Technology, Chinese Academy of Sciences, Shenzhen, 518055 Guangdong P. R. China; 4 Key Laboratory of Instrumentation Science and Dynamic Measurement, Ministry of Education, North University of China, Taiyuan, 030051 China; 5 Nano-Manufacturing Laboratory (NML), Shenzhen Research Institute of City University of Hong Kong, Shenzhen, 518057 China; 6 Department of Mechanical Engineering, City University of Hong Kong, Hong Kong SAR, Kowloon, 999077 Hong Kong

**Keywords:** Electrical and electronic engineering, Nanoscale materials, Nanoscale materials

## Abstract

Monitoring biophysical signals such as body or organ movements and other physical phenomena is necessary for patient rehabilitation. However, stretchable flexible pressure sensors with high sensitivity and a broad range that can meet these requirements are still lacking. Herein, we successfully monitored various vital biophysical features and implemented in-sensor dynamic deep learning for knee rehabilitation using an ultrabroad linear range and high-sensitivity stretchable iontronic pressure sensor (SIPS). We optimized the topological structure and material composition of the electrode to build a fully stretching on-skin sensor. The high sensitivity (12.43 kPa^−1^), ultrabroad linear sensing range (1 MPa), high pressure resolution (6.4 Pa), long-term durability (no decay after 12000 cycles), and excellent stretchability (up to 20%) allow the sensor to maintain operating stability, even in emergency cases with a high sudden impact force (near 1 MPa) applied to the sensor. As a practical demonstration, the SIPS can positively track biophysical signals such as pulse waves, muscle movements, and plantar pressure. Importantly, with the help of a neuro-inspired fully convolutional network algorithm, the SIPS can accurately predict knee joint postures for better rehabilitation after orthopedic surgery. Our SIPS has potential as a promising candidate for wearable electronics and artificial intelligent medical engineering owing to its unique high signal-to-noise ratio and ultrabroad linear range.

**Figure:**
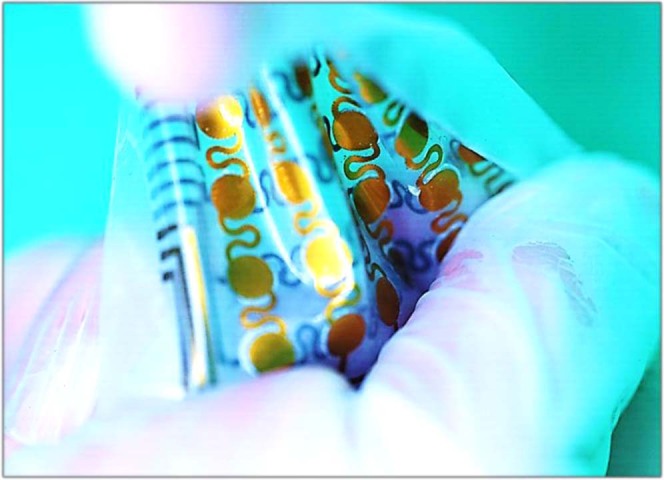
An ultrabroad-linear range (1 MPa) iontronic pressure sensor with superior sensitivity (12.43 kPa^-1^) and stretchability (up to 20%) was proposed for biophysical monitoring and deep learning-based knee-rehabilitation training.

An ultrabroad-linear range (1 MPa) iontronic pressure sensor with superior sensitivity (12.43 kPa^-1^) and stretchability (up to 20%) was proposed for biophysical monitoring and deep learning-based knee-rehabilitation training.

## Introduction

Wearable flexible pressure sensors (FPSs) that can continuously monitor biophysical information are urgently needed to provide early warning and rapid rehabilitation for fitness and healthcare^1–5^. Although important progress based on various sensing types, such as piezoresistive^6–10^, piezocapacitive^11–14^, piezoelectric^15–17^, and triboelectric effects^18–21^, has been achieved, FPSs with both ultrahigh linear sensing range (near 1 MPa) and sensitivity (over 10 kPa^-1^) are rare. Continuous monitoring of the human body requires a highly practical durability for FPSs, especially during physical exercise or emergency circumstances such as sudden falls, which can result in high pressure on the sensor. Therefore, there is an urgent need for sensors to not only possess an ultrabroad sensing range but also maintain acceptable sensitivity.

To address this obstacle, iontronic piezocapacitive (IPC) sensors with a high signal-to-noise ratio and broad sensing range have recently attracted attention^22–27^. Benefitting from their electrical double-layer (EDL)-induced high capacitance compared to traditional planar plate capacitors, their capacitance value increases accordingly with increasing applied pressure because of the enlarged accessible surface area and decreased ion transportation distance between two opposite electrodes. For instance, Pan et al. introduced an IPC sensor with a higher sensitivity and wide sensing range using an elastic ionic–electronic interface^25,28^, providing an ultrahigh unit-area capacitance. Guo et al. further achieved a graded intrafillable architecture-based iontronic sensor with an unprecedentedly high sensitivity (Smin>220 kPa^−1^) and a broad pressure regime (0.08 Pa-360 kPa) through the design of a new structural configuration of the IPC sensor to increase the spatially alternating ion transportation in nanometer spacing^29^. However, stretchable iontronic pressure sensors (SIPSs) with high sensitivity and a broad sensing range are rare. Given the circumstances employing a sensor when tracking a biophysical signal for a subject during an emergency, such as the considerable force impacts applied on the sensor during physical activities, it is necessary to improve the stretchability and operating range of the sensor.

In addition, this is expected to better monitor biophysical signals and facilitate patient rehabilitation after clinical surgery when combined with deep learning^4,5,30–36^. One typical strategy is to classify and predict perceptions with learning and training in the in-sensor deep-learning mode. For example, Yeo et al. developed a wireless soft electronic system and a data classification deep learning algorithm to provide a feasible tool for the real-time detection of ocular motions and portable therapy^37^. Additionally, Jan et al. used in-sensor adaptive learning to endow a wearable surface electromyography biosensing system with the capacity of accurate hand gesture recognition^32^. The above results demonstrated that deep learning has potential in the detection of biophysical monitoring by flexible wearable electronics.

Herein, we developed an on-skin SIPS with an ultrabroad linear working range to monitor various biophysical features and implemented in-sensor dynamic deep learning for knee rehabilitation training. Benefitting from stable conductive composite serpentine interconnected electrodes and the EDL capacitance effect, the SIPS achieved an ultrabroad linear sensing range (1 MPa) and enhanced sensitivity (12.43 kPa^−1^), as well as ultrahigh-pressure resolution (6.4 Pa). Furthermore, our sensor is able to monitor various features from biophysical signals to high foot pressure in a noninvasive, fast, and real-time manner. Additionally, owing to the ultrabroad sensing range, the sensor can still work well when subject to consecutive hammering tests imitating a demonstration of sudden impact during physical exercise or emergency circumstances such as a sudden fall. The neuro-inspired fully convolutional network (FCN) algorithm learns and trains the in-sensor data acquired from the SIPS with a processing circuit for evaluating the knee-motion state process. Such a strategy provides a favorable artificial intelligence (AI) assessment method for knee rehabilitation after orthopedic postoperative surgery, demonstrating the potential of a sensitive SIPS for clinical engineering.

## Results and discussion

### Overall device design and working principle

**Fig. 1:**
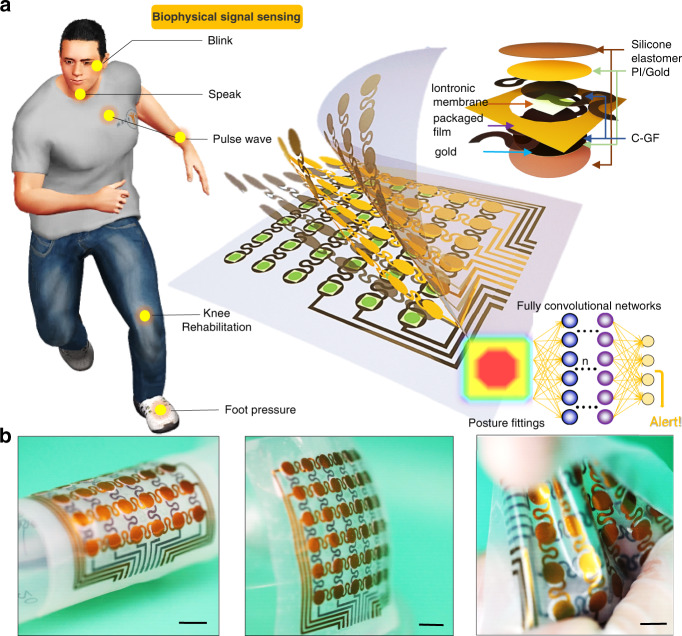
Design and special morphing capability of the SIPS. **a** Exploded view of the iontronic-capacitive sensing element composed of stretchable conductive C-GF electrodes, an ionic membrane, and a packaged silicone elastomer. **b** The device still retains its original integrity even under mechanical deformation, such as bending, stretching, or twisting (scale bar: 1 cm)

The exploded view illustrates that our designed system is primarily composed of an encapsulated soft silicone layer, a serpentine polyimide (PI) substrate coated with gold and carbon-graphite flake (C-GF) composite materials as electrodes, and a nanostructured ionic membrane (Fig. 1a). This thin, lightweight, and stretchable pressure sensor device allows for conformal attachment on the epidermal skin surface to accurately monitor biophysical signals such as eye blinks, throat vibrations, pulse waves, and other physical activities. As demonstrated in Fig. 1b, the wearable SIPS array enables intimate conformality with various nonplanar surfaces, such as a cylindrical and spherical nondevelopable surface. In addition, the device can bear severe twisting and stretching, exhibiting good potential for intimate skin wearability. Notably, ultrahigh-pressure produced during strenuous exercise or emergency circumstances (i.e., a sudden fall) can cause permanent damage to the sensors, which thus requires the sensor to provide an ultrahigh working range. Benefitting from the unique iontronic sensing mechanism of the sensor, an ultrabroad linear working limit of 1 MPa was achieved, which significantly broadens its applications. Furthermore, convolutional networks have been widely used recently to train and learn in-sensor modalities for systematic judgment of devices, which leads to more accurate decision-making or prediagnosis, such as robot awareness and rehabilitating management. FCN-aided knee rehabilitation monitoring of the sensor was therefore carefully analyzed in this study.

**Fig. 2:**
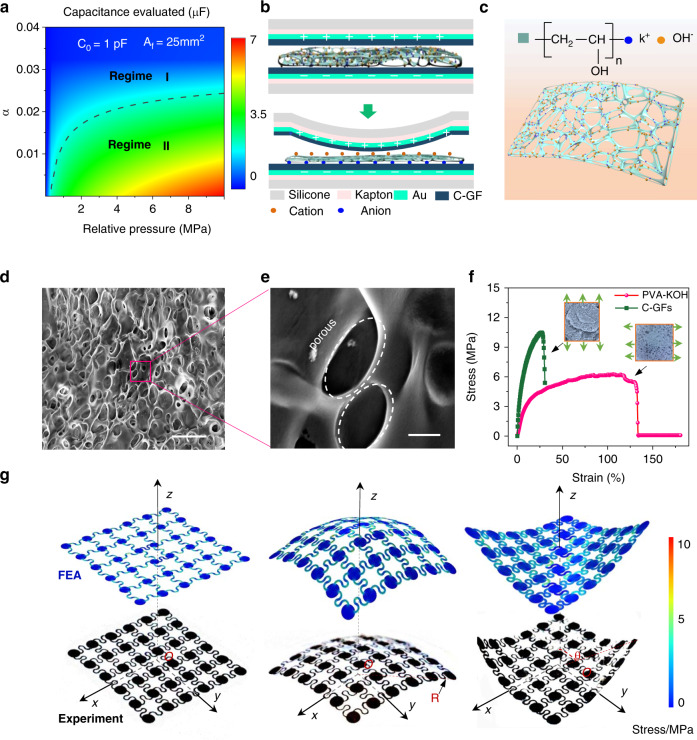
Working mechanism and material characterization of the sensor. **a** Evaluated capacitance by EDL equivalent calculation. **b** Schematic illustration of the sensor’s working principle under applied pressure. **c** Iontronic membrane model with the fiber-like PVA distribution. **d**–**e** SEM of the PVA-KOH ionic film and its corresponding locally enlarged image (scale bar: 20 μm and 2 μm, respectively). **f** Tensile tests of the conductive composite and ionic film. **g** Experimental and 3D-FEA results of the SIPS in three mixed modes: biaxial stretching, conforming to a hemispherical surface, and diagonal tensile

Based on the classic compression theory of porous fibrous assemblies^38^, the iontronic capacitance of the IPC sensor can be depicted by Equation

1 $$C = c_0 \cdot A\left( {\frac{P}{{\alpha \cdot E}} + A_{f_0}^3} \right)^{\frac{1}{3}}$$

where *c*_0_ is the initial capacitance, *A* is the effective sensing area, *P* is the applied pressure, E is the material equivalent elastic modulus, A_*f*0_ is the initial area fraction at *P* = 0, and α is the ionic composite distribution factor. The sensor’s capacitive characterization is developed with different p and α when *c*_0_ = 1 pF and A_*f*0_ = 25 mm^2^ in Fig. 2a^,^ which shows two obviously different digital capacitance areas: Regime I, which is usually applied in a conventional IPC sensor with a lower capacitance saturation, and Regime II, which is relatively desirable since sensor customization with fewer sensing materials can also develop a higher capacitance. Within a fixed applied pressure range, a higher capacitance variation level determined a higher sensitivity. Hence, the desirable capacitive level makes Regime II more favorable for SIPS construction. In this study, a polyvinyl alcohol (PVA)-KOH ionic film-based EDL iontronic sensor, which belongs to this region, is introduced. In contrast to several EDL sensing materials and structures^25,39–41^, the fibric-like PVA-KOH-based sensor also displays superior properties in the working range, sensitivity, and response time (Table S1). To prepare the device’s electrode, a fast, novel, and low-cost subtractive manufacturing approach was employed (Fig. S1). Arbitrary complicated patterns or electrode arrays can be easily manufactured, as shown in Figs. S2, S3, through this method. The detailed fabrication process can be found in the experimental section and SI. In addition, the dielectric layer also plays an important role in the capacitance level of the sensor device (Fig. 2b). When a certain pressure was applied on the SIPS, the ions inside the ionic film moved to opposite electrodes to form EDL capacitance, and the corresponding equivalent circuit is depicted as Fig. S4, resulting in a largely increased parallel capacitance formation compared to the traditional capacitive sensor. In this work, the soft and stretchable PVA-KOH ionic film was regarded as the stretchable dielectric layer (Fig. 2c and Fig. S5). Abundant ~10 μm pore nanostructures are beneficial for enhancing the capacitance due to the enhanced contact area under applied high pressure (Fig. 2d, e and Fig. S6). Furthermore, the PVA-KOH and C-GF films on the substrate film showed larger strains of 133% and 25%, respectively, as shown in Fig. 2f, both demonstrating their characteristics for stretching electronics. For a quantitative analysis of the stretching and conformal performances of the SIPS, finite element analysis (FEA) and corresponding experimental characterization were conducted (Fig. 2g, movies S1–S3). A planar biaxial, spherical tight conformal (*R* =8 cm), and diagonal stretching tensile test were introduced here. Symmetrical loading means of the stress in experimental tests provide guides for FEA simulations and enable the experimental result to highly agree with the simulation result. All FEA results illustrate the tensile strain concentration in each serpentine connection, which is consistent with the mechanical properties of serpentine structure deployments in experimental tests. A minimum deformation of 20% was experimentally observed in the diagonal stretching test when the device reached its limit stress of 9 MPa, and Fig. S7 further exhibited a deformation of over 20% of the SIPS protected by silicon elastomer under uniaxial tensile tests, which meets the on-skin device’s requirements^42^. Additionally, Figs. S8–S10 and movies S4, S5 exhibit the desirable deformation capability and conductivity of the well-aligned serpentine electrodes under large tensile strength, which revealed the electrode’s excellent stability and feasibility.

### Device characterizations

**Fig. 3:**
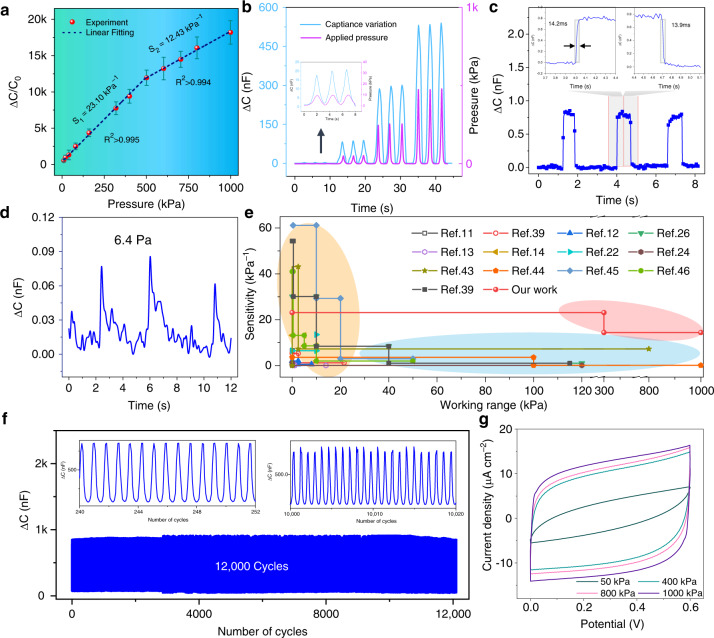
Electronic performance of the sensor for pressure sensing. **a** Normalized change in the experimental capacitance versus the applied pressure. **b** Capacitive response versus different applied stresses. **c** Outlined response and relaxation time under a pressure of 5 kPa. **d** Detecting stress resolution at low pressure. **e** Comparison of the sensitivity of our ionic sensor with other sensors. **f** Long-term durability over 12000 cycles under a pressure of 800 kPa. **g** Current density curves versus the potential under independently applied stress

The performance of the SIPS unit was analyzed as shown in Fig. 3. The sensitivity (S) of the sensor is typically defined as S = δ(ΔC/C0)/δ(ΔP), where C0 is the initial capacitance without any applied pressure and Δ*C* and Δ*P* are the variations in the capacitance and corresponding applied pressure, respectively. The ΔC/C0 versus the applied pressure is plotted as shown in Fig. 3a. Our SIPS sensor exhibits an ultrabroad sensing range up to 1 MPa while still showing superior sensitivity of 23.10 kPa^-1^ (below 325 kPa) and 12.43 kPa^-1^ (325 kPa to 1 MPa). Additionally, a highly linear relationship between the produced capacitance value and pressure variation was plotted, which benefitted from the EDL capacitance effect and the stable conductive composite-based serpentine interconnect electrodes. Such a superior trade-off between the sensitivity and working range, to our scientific knowledge, has never been reported before and would provide extended practical applications from low pressure (such as pulse or respiration) to high pressure (e.g., falling impact or foot pressure). Control experiments that used pure polydimethylsiloxane (PDMS) and copper as the dielectric layer and electrode (Fig. S11), respectively, showed inferior sensitivities of only 0.0424 kPa^-1^ and 0.0002 kPa^-1^. Such high performance is mainly derived from the high surface areas, good conductivity of the added graphite flakes (GFs) into the carbon ink (Figs. S12, S13), and synergistic reaction between the ionic dielectric layer and the multimaterial composition. Moreover, Fig. 3b exhibits a nearly negligible hysteresis between the capacitance variation and applied pressure, demonstrating the excellent linear response nature of our sensor. In addition, our sensor exhibited a rapid response time of 14.2 ms and relaxation time of 13.9 ms under a continuous pressure of 5 kPa (Fig. 3c), a high signal-to-noise ratio of 7.68 and a minimum pressure detection limit of only 6.4 Pa (Fig. 3d), achieving a great balance between the detection limit and sensing range. This was also demonstrated by a comparison of our study with other related reports (Fig. 3e**)**, which showed an ultrabroad sensing range and favorable sensitivity comparable and even superior to other works^11–14,22,24,26,39,42–46^. Long-term durability determined its practical capability. Even under a high pressure of 800 kPa or a low pressure of 10 kPa over 11000 cycles, our sensor holds a constant value without obvious signal decay (Fig. 3f, Fig. S14). To further elucidate the sensing mechanism, electrochemical testing of the current density versus the operating potential variation of our sensor under different pressures and scanning rates is shown in Fig. 3g and Fig. S15, respectively. Obviously, the cyclic voltammetry (C-V) curves exhibit a rectangular shape and typical EDL capacitive performance. The integrated profile area also increases with the applied pressure, further demonstrating its supercapacitive mechanism. The effect of thickness and frequency on capacitance was also investigated, as shown in Fig. S16.

### Vital biophysical monitoring

**Fig. 4:**
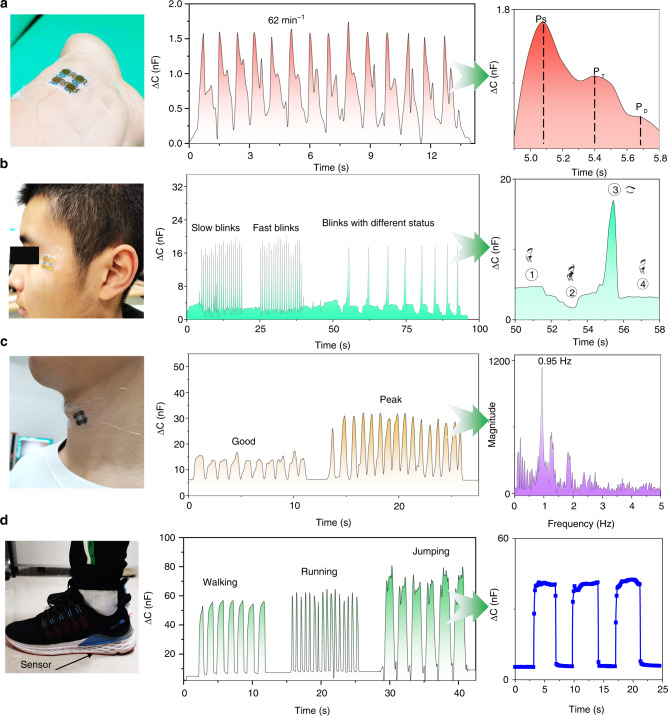
Biophysical signal monitoring of an adult male (178 cm in height, 70 kg in weight). **a** Pulse monitoring of the SIPS unit wrapped on the wrist and its corresponding pulse wave peaks for cardiovascular disease detection. **b** Blink evaluations while the SIPS unit is conformal with the corner of the eye with distinct opened states and different goggle angles. **c** Records of the sound spectrum while saying different words and the corresponding sound frequency spectrum by Fourier transformation. **d** Information from three motion features and their corresponding response times under walking mode while sensor withstands foot pressure

To further demonstrate the practical application of the sensor by ultrabroad working range features, various noninvasive, fast, and real-time biophysical monitoring was conducted, as shown in Fig. 4. Typically, pulse, eye blinks, throat vibrations, and plantar pressure are important indicators for various disease diagnoses or life monitoring. As illustrated in Fig. 4a, the capacitance fluctuations of our sensor accurately recorded the pulse wave for the volunteer. A heartbeat frequency of 62 min^-1^ within the standard of a normal person’s pulse rate between 60 and 80 min^--1^ was observed, and three obvious peaks, referred to PS (percussion), PT (tidal), and PD (diastolic) waves, as important clinical signals of cardiovascular disease, can also be distinguished. Additionally, our sensor is able to detect eyeblinks with different statuses and frequencies owing to its excellent sensitivity and fast response time (Fig. 4b), which is beneficial for the early evaluation of microexpressions or eye disease. Similarly, throat vibrations with various word spellings were quantitively analyzed (Fig. 4c). The speaking magnitude and frequency were monitored via Frontier transformation with a dominant frequency of 0.95 Hz, indicating good voice recognitions. Finally, dynamic plantar pressure was also successfully recorded when walking, running, and even jumping. The capacitance vibrations of frequency and magnitude accurately represent motion characteristics of corresponding postures, indicating their application in motion prediction (Fig. 4d).

### Anti-impact application

**Fig. 5:**
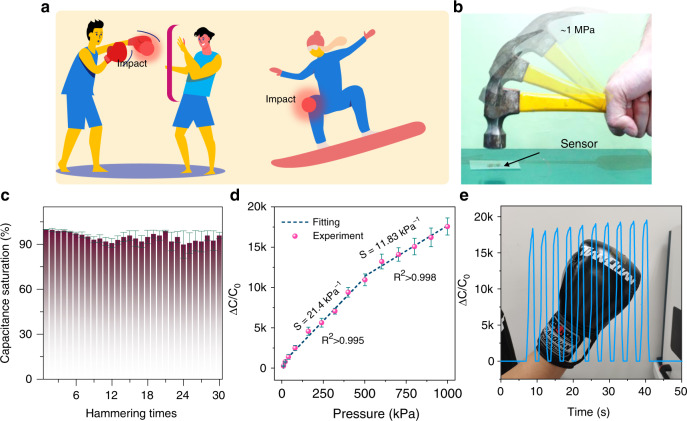
Consecutive hammering tests. **a** Some extreme sports, such as boxing and skiing, that potentially generate high impact force. **b** Optical image of the sensor subject to consecutive sudden strikes by a hammer (0.25 kg). **c** Capacitance retention histogram under continuous and stable strikes. **d** Capacitance change with the pressure of the SISP after the hammering strike test. **e** Capacitance response test of the SISP under continuous boxing strikes

To deeply exploit the advantage of the ultrabroad linear sensing range of the flexible sensor, its application scenario is shown in Fig. 5. An increasing number of people are now participating in extreme sports such as boxing and skiing to stay healthy. However, during these exercises, some unavoidable/unexpected situations often occur, such as falls, bruises, collisions and so on (Fig. 5a). Under these accidental circumstances, a large impact force can cause permanent damage to the flexible sensor if the sensing range of the sensor is relatively low. To verify the SIPS’s application in severe high-pressure situations, our sensor was subjected to consecutive hammering tests, which can imitate a demonstration of sudden impact (Fig. 5b, the transient violent hit pressure is estimated to be over 1 MPa). It is obvious that the capacitance saturation remained over 90%, and the sensitivity and working range remained almost unchanged, even after 30 instantaneous impacts with dynamic violent hammer strikes (Fig. 5c, d). In addition, a transient boxing impact test was conducted under a stress level near 1 MPa while the sensor was conformal with the end surface of a boxing glove. A male volunteer wearing a boxing glove suddenly struck the wall with 10 continuous strikes. The sensor response almost achieves capacitance saturation and remains stable (Fig. 5e), further verifying the sensor’s durability and revealing that our sensor can meet practical ultrahigh-pressure needs.

### Deep learning-aided knee rehabilitation

**Fig. 6:**
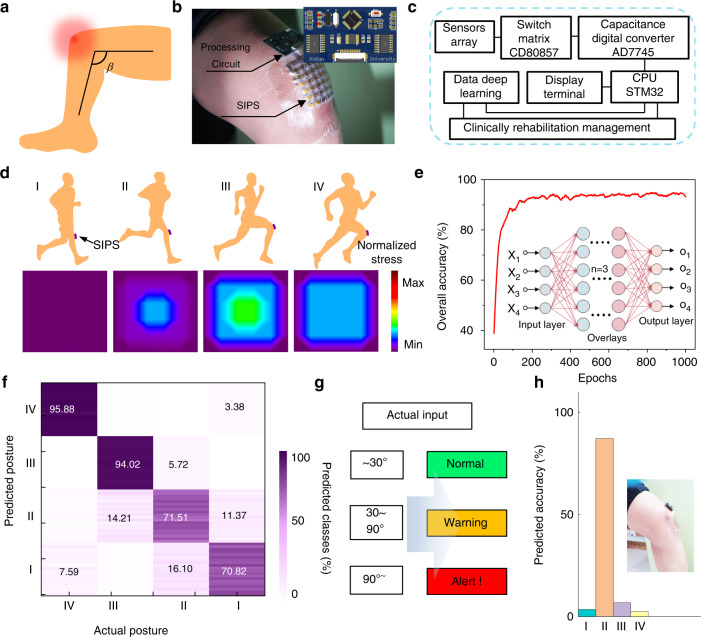
Applications for knee rehabilitation monitoring. **a** Knee-posture illustration of personal sports injuries. **b** Optical image of the SIPS connected with a processing circuit mounted on a participant’s knee. **c** Functional block diagram of multichannel capacitive signal acquisition. **d** Normalized kneecap stress distribution measured from four bending statuses. **e** Whole accuracy of acquiring kneecap stress data after 1000 epochs of deep learning based on the FCN method. **f** Classification confusion matrix for actual posture prediction. **g** Schematic diagram of presupposed bending angle categorizations. **h** Verification of acquiring data from special knee bending status in our deep-learning system

Deep learning was recently extensively studied with flexible sensors to broaden its potential application. To demonstrate its application in biophysical monitoring and health care applications, the rehabilitation of knee joints aided by a sensor is evaluated. Overflexed or extended states can lead to meniscus injuries in the knees (Fig. 6a). The inherent pressure-level and position monitoring of knee joint postures therefore acts as a key symptom to evaluate rehabilitating conditions after orthopedic surgery to avoid further injury during clinical rehabilitation training. When the integrated SIPS with the processing circuit was fixed on the volunteer’s knee joint (Fig. 6b), the stress distribution of the kneecap could be recorded smoothly via the capacitance acquisition circuit transmitting to the storage chip. The functional block diagrams of the processing circuit in Fig. 6c show the systemic flow of the capacitance to the digital voltage transformation and main control chip for switching and scanning among overall channel signals (see the circuit design in the Methods for details, Figs. S17, S18). As illustrated in Fig. 6d, four typical postures of the knee joint regarding unbending, slight bending, bow stance, and striding were defined as I, II, III, and IV postures, respectively. Hence, each normalized distributing pressure map represents the corresponding knee joint movement features, including the pressure level and position. For accurate prediction of knee joint postures, the neuroinspired FCN algorithm was conducted locally, where 80 arrays after the two-dimensional matrix to a one-dimensional array transformation were used for training and updating in the deep learning model, with 1000 epoch iterations during 3 overlayer training networks (see the detailed process of modeling deep learning in the Methods and Table S2). Each posture contains 36 numerical values in the iterative process. A schematic illustration of FCN-aided deep learning for knee posture prediction is shown in Fig. S19. In the FCN-aided deep-learning process, four posture predictions were obtained by training and iterating three overlays. The whole accuracy of the four postures I~IV was modified in Fig. 6e. The FCN-aided model exhibited a 91.8% high average training accuracy, demonstrating the deep-learning model’s high robustness and feasibility. The classification confusion matrix holds 80 times the posture recorded in the experiments (Fig. 6f), in which the text values are the accuracy percentages of the predicted posture. The diagonal colored background represents the predicted correct proportion of each class. Owing to the deep-learning mode training from postures I to IV, the systemic feasibility is cumulatively developed, so the later predicted accuracy is higher than foregoing classes, which render no interference on the overall evaluation. Finally, rather than evaluating the training posture, the focus on the actual posture feedback and prediction is more important for practical uses. Based on the bending angle (*β*, Fig. 6a) of the knee joint, three actual posture states can be defined as prompt categories of normal (~30°), warning (30°−90°), and alarming (90°~) in the rehabilitating training (Fig. 6g), and corresponding the actual knee bending angle of 180°, 90–180°, and ~ 90° referred as normal, as a warning, as alarming prompt, respectively. A patient’s knee posture can be an early warning of a corresponding status to awaken AI rehabilitation platforms by our SIPS. A specially tested posture as in the inset was inputted into the training mode, which proleptically distinguished it as the second class with 93.27% ultrahigh precision (Fig. 6h), exhibiting the excellent prediction capability of the in-sensor deep-learning mode in clinical knee rehabilitation prediagnosis.

## Conclusions

In summary, this work demonstrated a stretchable flexible iontronic pressure sensor with an ultrabroad linear sensing range up to 1 MPa while still presenting superior sensitivity, which benefitted from EDL capacitance and stable conductive composite-based serpentine interconnect electrodes. Furthermore, the overall mechanically soft feature, excellent ultrabroad linear range-sensitive properties, and high detection limit make our device intimately contact human skin seamlessly and successfully track biophysical signals such as pulse waves, muscle movements, and plantar pressure. The ultrabroad sensing range guarantees its superior anti-impact ability, even in the high-pressure range. Importantly, the SIPS enabled the detection of kneecap pressure distribution and successfully predicted the corresponding motion posture by a neuro-inspired FCN algorithm with learning and training in the in-sensor deep-learning mode. We hope that our sensitive SIPS has the potential as a favorable AI assessment for orthopedic postoperative knee rehabilitation or other health monitoring in future clinical engineering.

## Methods

### Fabrication of the serpentine interconnect electrodes and ionic film

To prepare the serpentine electrodes, a silicone elastomer (100 μm, Xingye Plastic Products Factory, Guangdong Province) was first laminated on a glass slide (10 × 10 cm^2^). A PI film (50 μm, Shenzhen Wangyang Adhesive Tape Co. Ltd) was then carefully adhered to the above-prepared silicone film. Chromium and gold film were subsequently sputtered onto the PI film by magnetron sputtering (Gewei, GVC-2000) for 60 s at a power of 100 W. Then, a composite ink was made by mixing graphite flakes (Shenzhen Suiheng Technology Co. Ltd), carbon nanoparticle ink (CH-8 (MOD2, Shitiao International Ink Co. Ltd, Japan), and slow drying diluent (Zhengxing Ink Co. Ltd, Wenzhou, Zhejiang) together under continuous stirring for 30 min with a weight rate of 1:20:20. Then, the ink was spun onto PI coated with gold at a low speed of 800 rpm for 10 s first and a high speed of 1600 rpm for 10 s through spin-coating. After the composite film was fully cured at 70 °C for 4 h, the designed pattern was obtained directly by a microcutter (Anycut, C16) at a speed of 50 m s^-1^ and 1% pressure.

The PVA-KOH gel ionic film was fabricated according to our previous research^47^.

Briefly, 3 g of PVA was initially dissolved in 20 mL of deionized water at 100 °C with vigorous stirring for 1 h, followed by the addition of 10 mL of a KOH solution at a concentration of 0.3 g mL^−1^. The PVA-KOH gel ionic solution was then poured onto sandpaper and cured at room temperature for 12 h. The conductive electrodes and the ionic film were then observed with a scanning electron microscope (SEM, JEOL JSM-6360LV).

### Assembly of the flexible iontronic device

The SIPS is mainly composed of a flexible packaging elastomer, nonmetal conductive stretchable electrodes, and ionic film. The detailed assembly process of the SIPS and of the corresponding connection pad are shown in Fig. S20. First, a fabricated ionic film was engraved by a 355 nm UV laser (30 kHz pulse frequency, 300 mm s ^−^^1^ cutting speed for 4 repetition cuttings) to obtain 36 uniform circular ionic substrates. Each independent circular ionic film was aligned with an electrode pad and cured at 50 °C for 0.5 h. Additionally, a 355 nm UV laser was used to cut the copper connection pad with a thickness of 10 μm for the device connection (50 kHz pulse frequency, 300 mm s ^−^^1^ cutting speed for 10 repetitions). The laser-based connection pad that was transferred with water-soluble tape (AQUASOL tape) was connected to the conductive composite pad with conductive silver paste and fixed on the bottom electrodes by a hot embossing machine (Changshang Chen e-commerce Co., Ltd). Subsequently, the top alignment with the bottom electrodes was connected together by van der Waals forces between two larger silicone elastomer films, followed by bonding at 70 °C for 24 h.

### Electromechanical and mechanical testing of the device

The capacitive properties were tested via an LCR digital bridge meter (TH2832, Tonghui Electronics Co. Ltd, Changzhou, and HIOKI IM2536). Compression or tensile tests were applied by a universal mechanical testing machine (Zhi Qu, ZQ-990B). Corresponding current and potential correlations of the sensor were directly recorded by the source meter (Keithley 2400) or electrochemical workstation (Chenhua, CHI760e) with a two-electrode mode.

### Analogical circuit design

Thirty-six channel sensing acquisitions were connected with two switching matrix chips (CD80857), with a row and column selection rate of 50 ms for each element capacitance data. The capacitance obtained was transmitted to a capacitance digital converter (AD7745) to obtain the corresponding voltage change. Then, each signal was processed with a marked output in the CPU control center (STM32).

### Modeling deep-learning process

Using stratified sampling in each category. Overall, 80 sets of four postures were trained with a three-tier structure by the FCN algorithm. The activation function adopts the Re-Lu activation algorithm. During the learning process, the batch size, learning rate, and weight attenuation were set to 4, 1e^−2^, and 1e^−1^, respectively. A total of 1000 epochs are trained using stochastic gradient descent. The training set accounts for 90%, and the test set accounts for 10%.

## Supplementary information

Supporting information-For publication

Consent Form

Movie S1

Movie S2

Movie S3

Movie S4

Movie S5

Graphical Abstract
