# Supplementary material for: Stretchable and anti-impact iontronic pressure sensor with an ultrabroad linear range for biophysical monitoring and deep learning-aided knee rehabilitation
Source: Microsyst Nanoeng. 2021 Nov 17;7:92. doi: 10.1038/s41378-021-00318-2 (PMC8599697; doi:10.1038/s41378-021-00318-2)
Supplement: Supplementary file 1 — Supporting information-For publication [file 41378_2021_318_MOESM1_ESM.pdf]

Stretchable and Anti-impact Iontronic Pressure Sensor with an Ultrabroad Linear Range for Biophysical Monitoring and Deep Learning-aided Knee Rehabilitation

Hongcheng Xu[1,2#], Libo Gao[1,2,#]*, Haitao Zhao[3#], Hanlin Huang[1], Yuejiao Wang[2],Gang Chen[1], Yuxin Qin[1], Ningjuan Zhao[1], Dandan Xu[1], Ling Duan[1], Xuan Li[1], Siyu Li[1], Zhongbao Luo[4], Weidong Wang[1,2]*, and Yang Lu[5,6]*

[1]School of Mechano-Electronic Engineering, Xidian University, Xian 710071, China

[2]CityU-Xidian Joint Laboratory of Micro/Nano-Manufacturing, Shenzhen 518057, China

[3]Materials Interfaces Center, Shenzhen Institutes of Advanced Technology, Chinese Academy of Sciences, Shenzhen 518055, Guangdong, PR China.

[4]Key Laboratory of Instrumentation Science and Dynamic Measurement, Ministry of Education, North University of China, Taiyuan 030051, China

[5]Nano-Manufacturing Laboratory (NML), Shenzhen Research Institute of City University of Hong Kong, Shenzhen 518057, China

[6]Department of Mechanical Engineering, City University of Hong Kong, Hong Kong SAR, Kowloon 999077, Hong Kong

#These authors contribute equally to this work.

*Author to whom correspondence should be addressed:

E-mail: lbgao@xidian.edu.cn; wdwangcn@xidian.edu.cn;yanglu@cityu.edu.hk

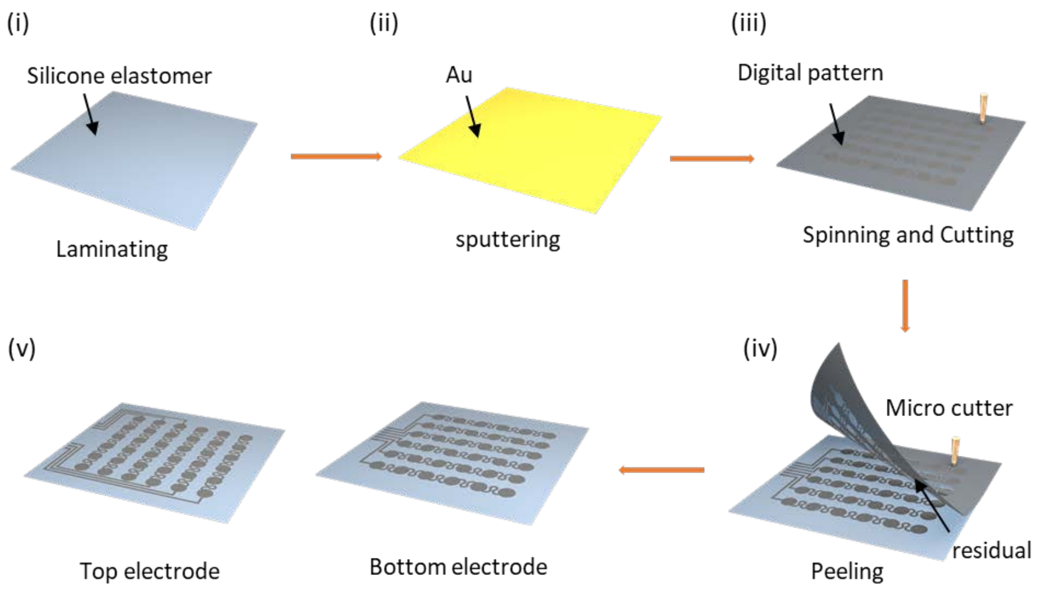

Figure S1. Manufacturing process of the bottom and top C-GFs array by micro-cutting technology.

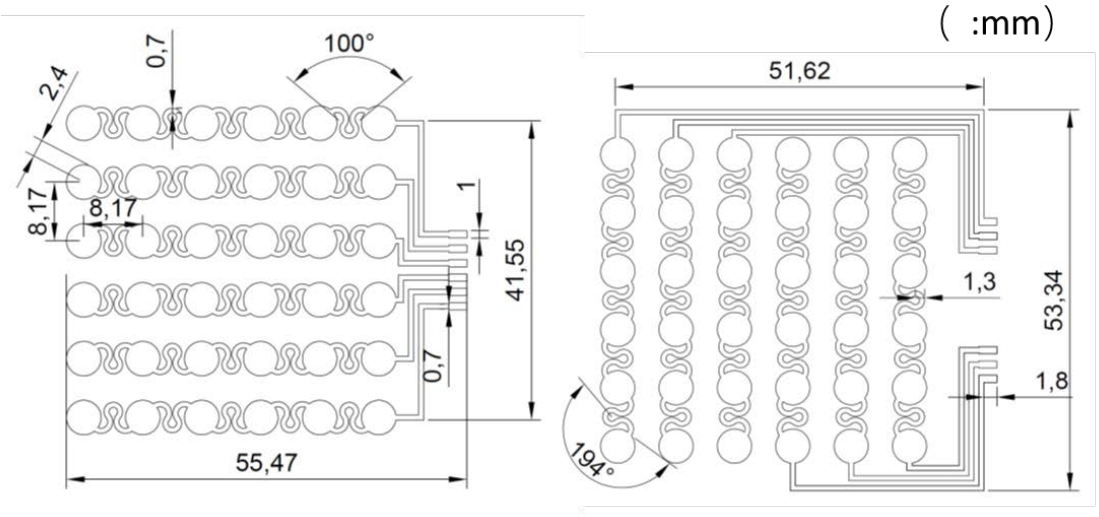

Figure S2. Geometric dimension of the bottom and top electrodes detailly.

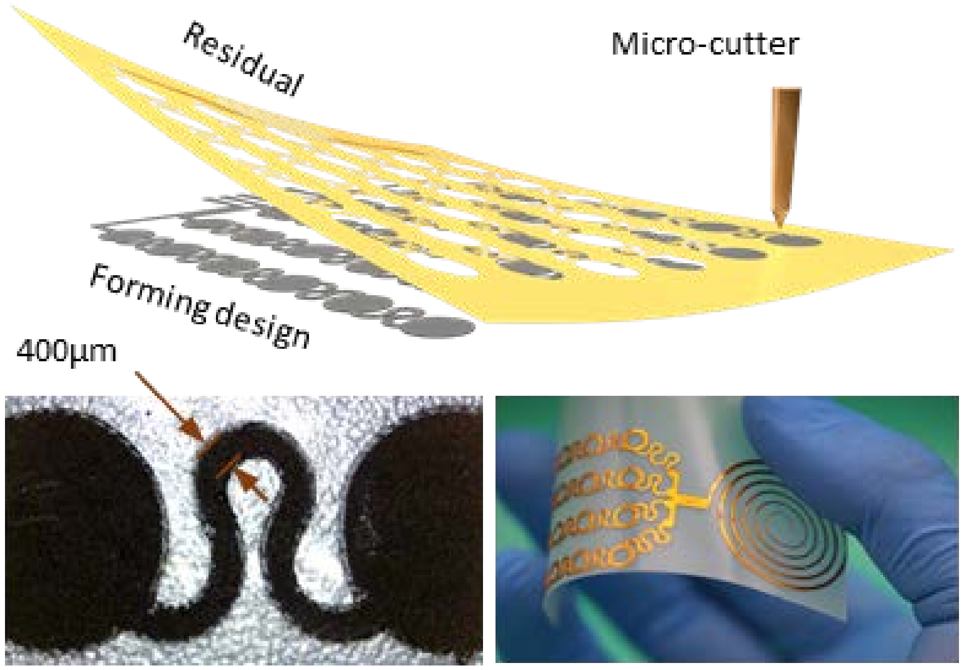

Figure S3. Schematic illustration of the micro-cut and paste processing for C-GFs electrodes and the application in metal architecture.

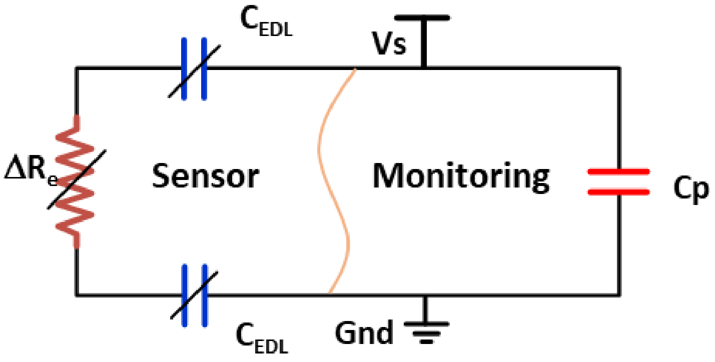

Figure S4. Schematics depiction of the EDLs equivalent circuit.

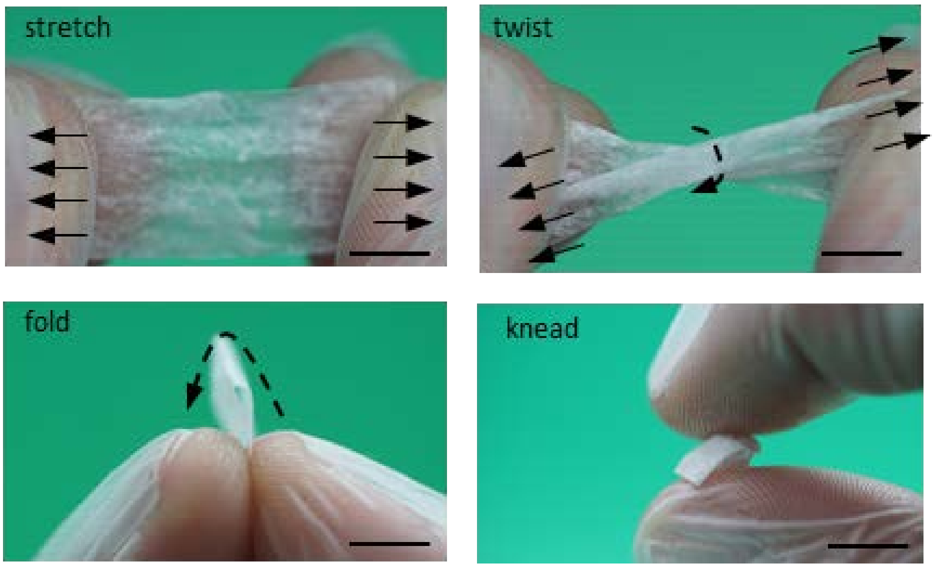

Figure S5. Optical images of processing the fabricated ultra-thin ionic film (20 μm) under four distinct modes (scale bar: 5 mm)

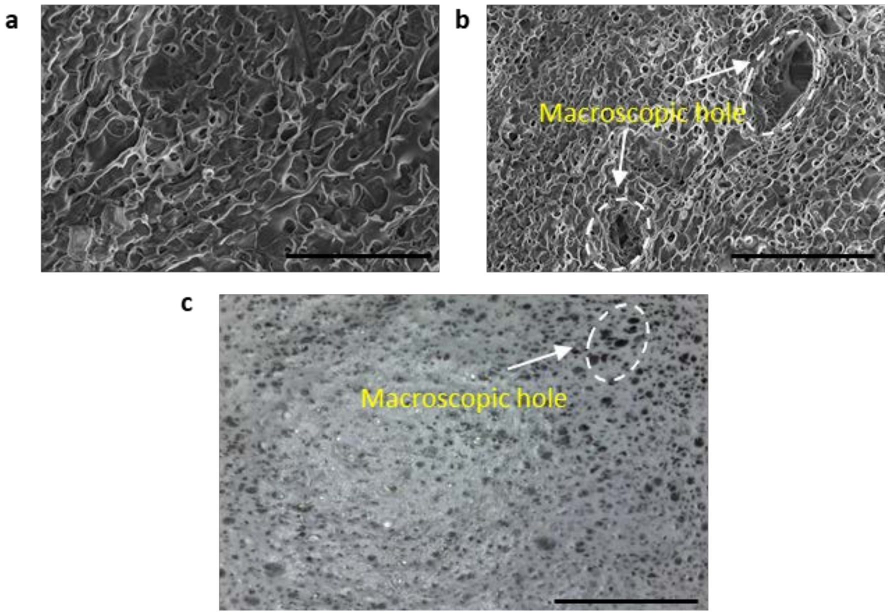

Figure S6. **a** and **b**. SEM of of the PVA-KOH ionic film and corresponding local enlarged image (scale bar: 50 μm and 30 μm, respectively). **c**. Optical image of the stretched ionic film (scale bar: 1 mm).

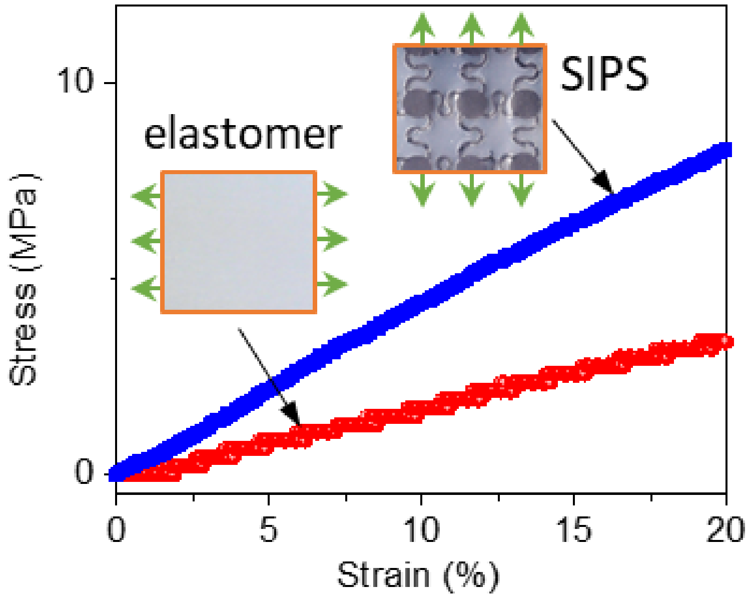

Figure S7. Uniaxial tensile tests of the whole sensor and silicone elastomer for verification in deformation capability.

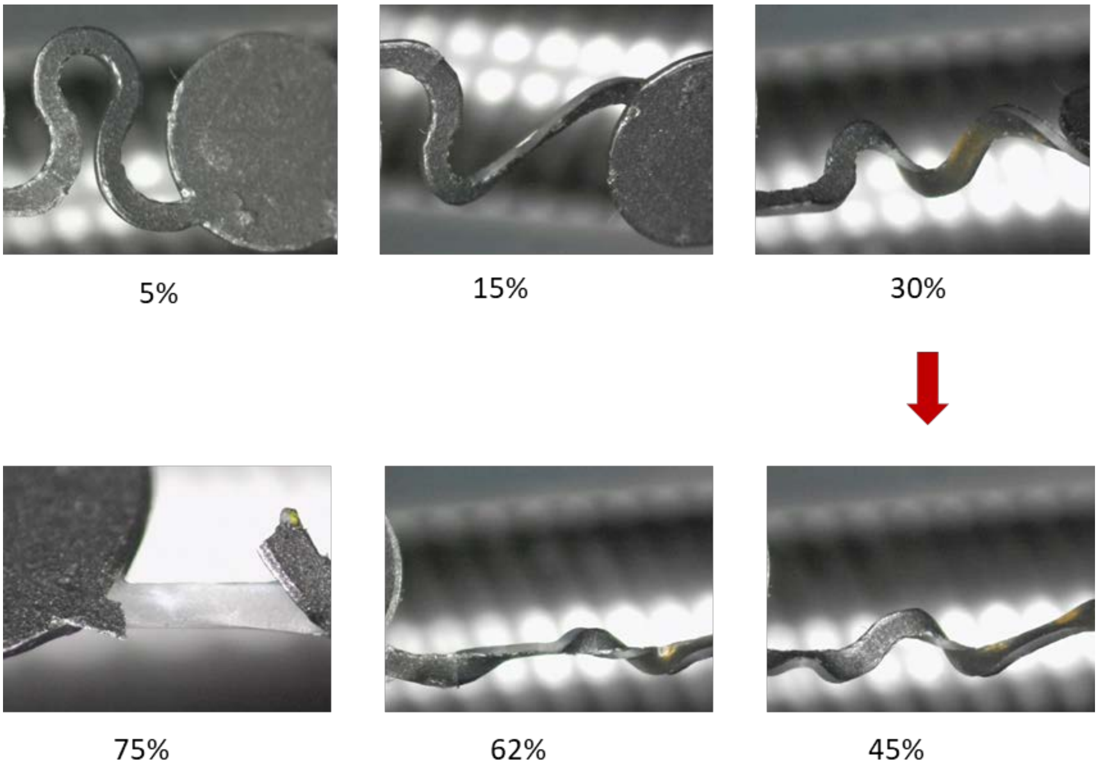

Figure S8. In-situ experimented images of serpentine electrodes with different tensile status.

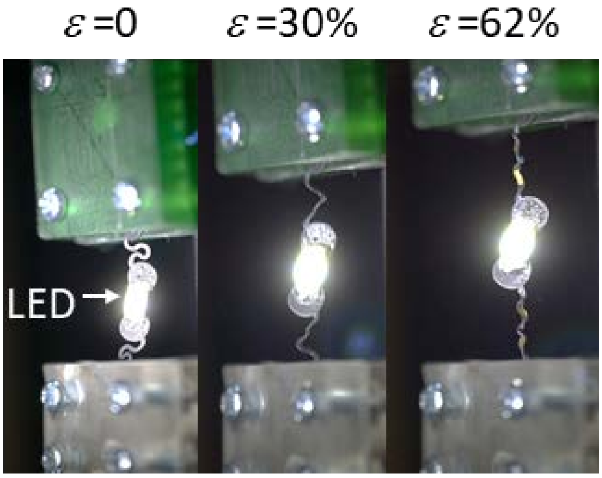

Figure S9. LED connected with serpentine electrodes that are stretched with deformation to 62%, verifying the excellent tensile property.

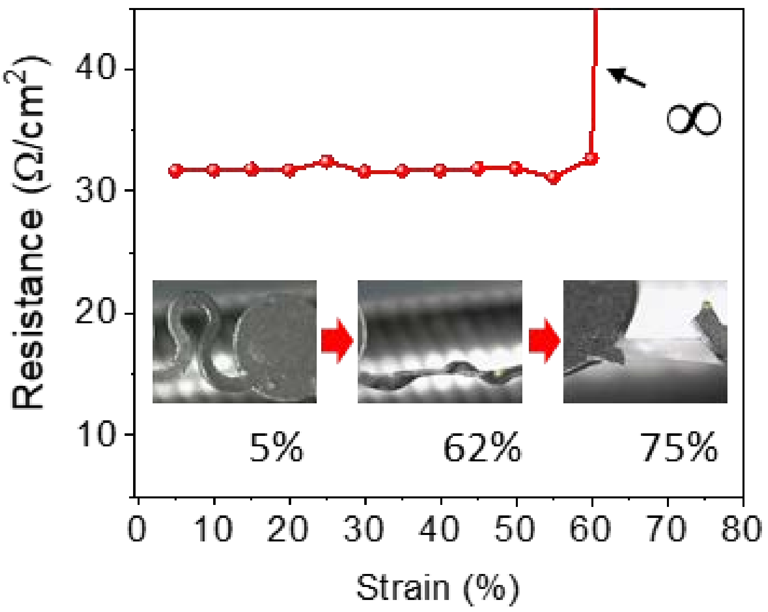

Figure S10. Square resistance of the serpentine metalloid electrodes in situ tensile tests.

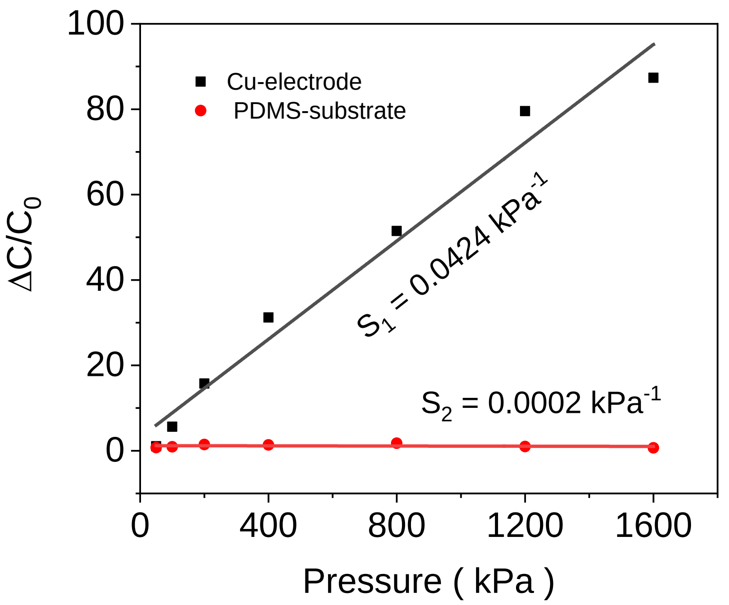

Figure S11. Comparison tests of the normalized capacitive transformation of the sensor with cupreous electrodes and PDMS-substrate, respectively

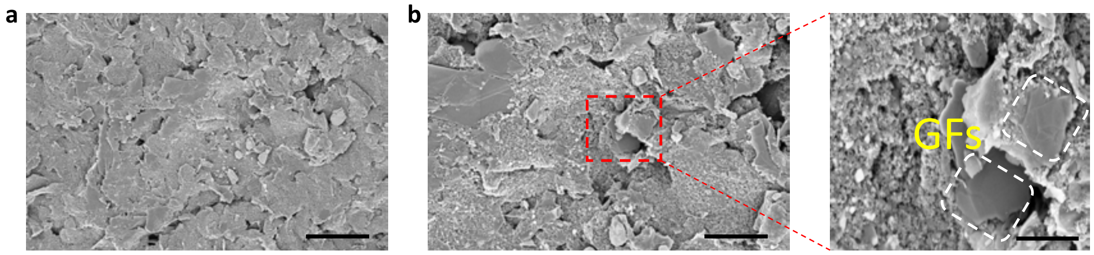

Figure S12. **a** and **b** SEM of the conductive C-GFs composite (scale bar: 25 μm, 10 μm, and 3 μm, respectively).

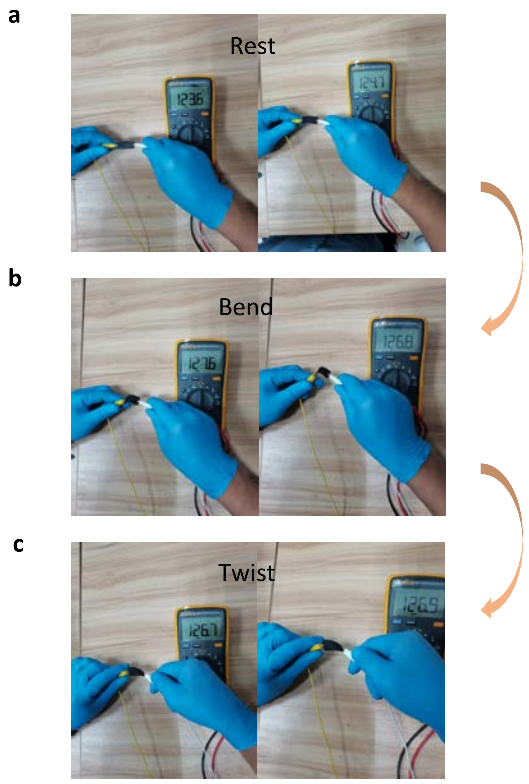

Figure S13. Resistance of the conductive C-GFs composite at **a** rest, **b** bending and **c** twisting status.

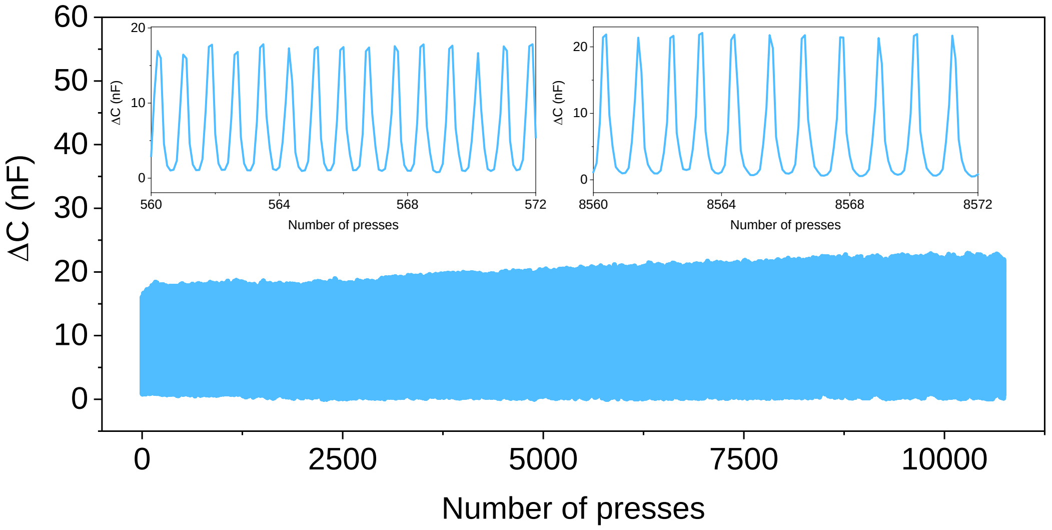

Figure S14. Durability test of the sensor under lower applied pressure of 10 kPa for 11000 cycles.

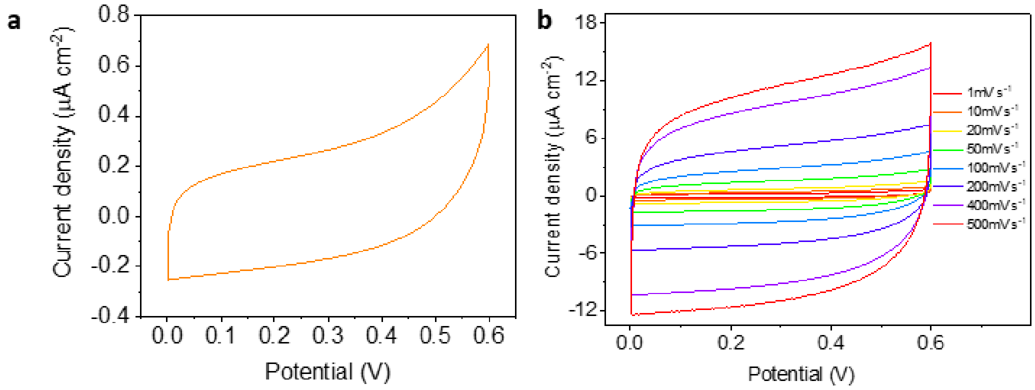

Figure S15. Current density curves versus the potential. **a**. under the pressure of 0 kPa, **b**. under different scanning speed.

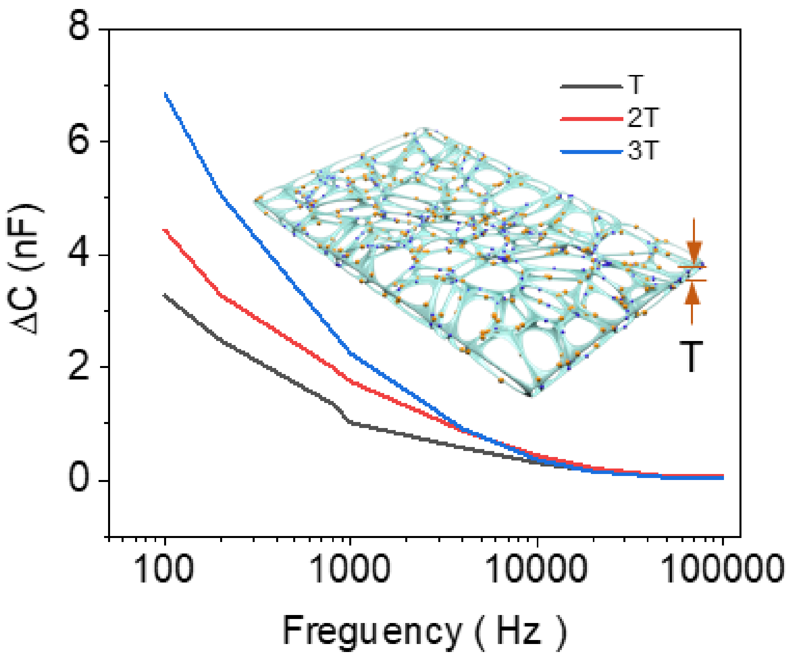

Figure S16. Capacitive variations versus measured frequency when the thickness of the ionic film is transformed. As a demonstration, the frequency also effects influence the capacitive properties. The result showed the frequency spectrum feature with different thicknesses of the ionic film (T=115 μm). A clear opposite correlation of frequency and the capacitance accords with the typical LC resonance rationale.

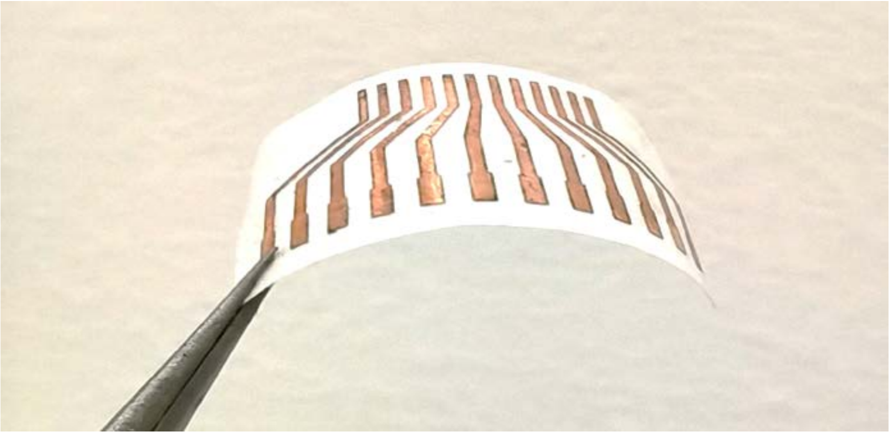

Figure S17. Laser engraved electrode between the device and circuit.

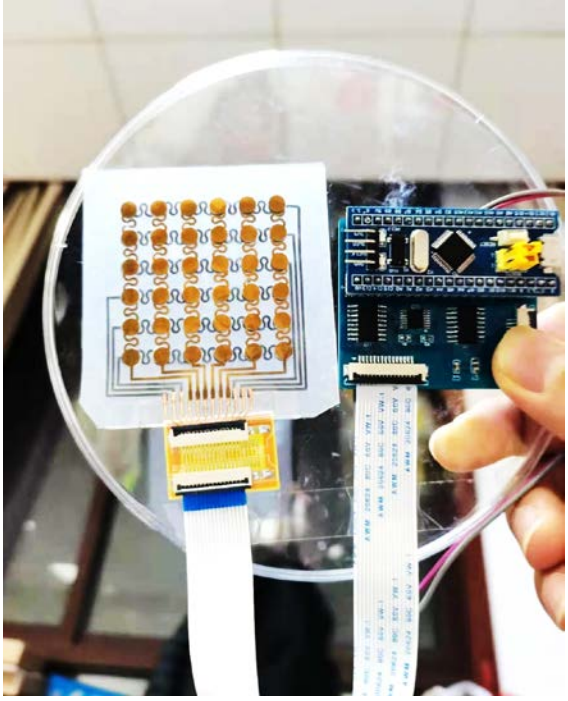

Figure S18. Optical image of the conformal device connected with the array-signals acquisition and process circuit.

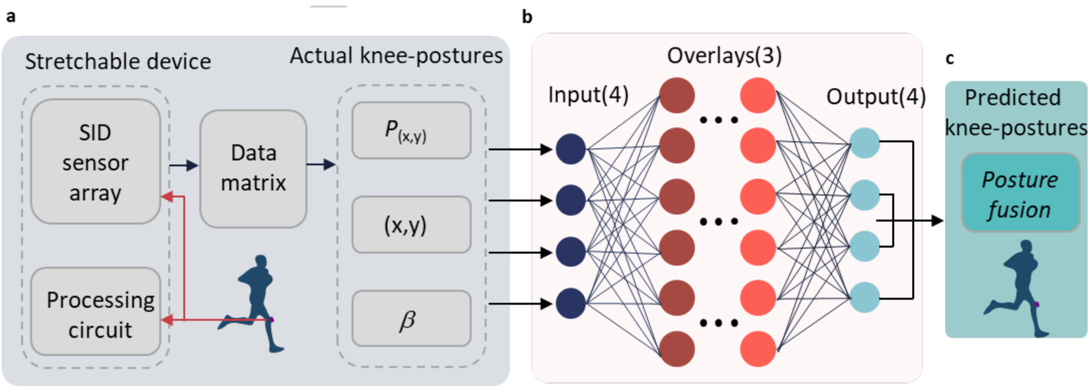

Figure S19. Schematic illustration of the FCN aided deep-learning for knee posture prediction. **a** Actual knee-postures was primarily detected by our SIPCS connected with a processing circuit. **b** FCN aided deep-learning model with three overlays for four postures predictions. **c** Final predicted posture based on the training accuracy from the deep-learning model.

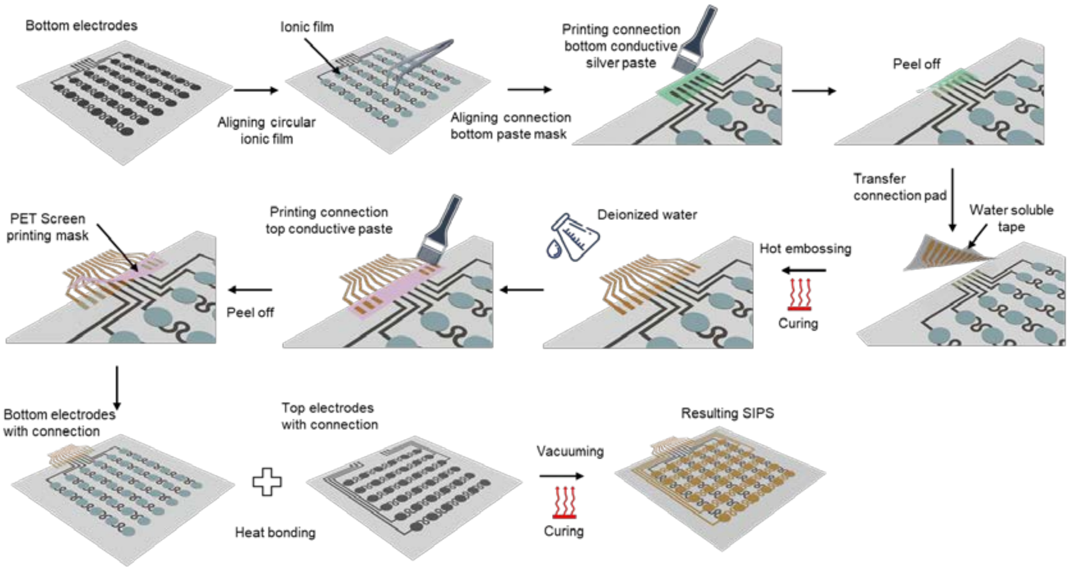

Figure S20. The assembly process of the SIPS and the corresponding connection pad.

Table S1. Comparisons of the ionic sensor with different EDLs materials and structures.

	Structure model	Ionic material	Sensitivity	Working range	Response time
Ref. 40	Droplet-contact	[EMIM]$^{+}$[BF4]$^{-}$	31.1 kPa^{-1}	<6 kPa	78 ms
Ref. 25	Polymeric matrix	Nafion	5 kPa^{-1};	<5 kPa	
			0.15 kPa^{-1}	<30 kPa	
Ref. 41	Fiber-like	PEDOT@PVDF	18.4 kPa^{-1}	<100 Pa	15 ms
			0.7 kPa^{-1}	<12 kPa	
Ref. 39	Elastic microstructure	[EMIM]$^{+}$[TFSI]$^{-}$@PVDF-HEF	54.31 kPa^{-1}	<500 Pa	29 ms
			1.03 kPa^{-1}	<115 kPa	
Our work	Fiber-like	PVA-KOH	23.10 kPa^{-1}	**<325 Pa**	**14.2 ms**
			**12.43 kPa^{-1}**	**<1 MPa**	

Table S2. Predicted accuracy of different knee joint postures based on experiment acquiring data.

Posture I	0.77475819	0.138477	0.016417	0.070348
	0.768707	0.155473	0.016099	0.059721
	0.71764567	0.195628	0.022515	0.064211
	0.76415567	0.164493	0.015994	0.055357
	0.72336141	0.147713	0.026396	0.10253
	0.70995645	0.192738	0.02478	0.072525
	0.76390039	0.147972	0.017832	0.070296
	0.71062906	0.167014	0.028059	0.094298
	0.73655313	0.189633	0.019054	0.054761
	0.71685299	0.171621	0.026124	0.085402
	0.69449445	0.143331	0.032829	0.129346
	0.78932772	0.157272	0.01147	0.04193
	0.71810631	0.207294	0.020573	0.054027
	0.73769796	0.163284	0.022103	0.076915
	0.77269998	0.116245	0.017593	0.093462
	0.71912435	0.172653	0.02515	0.083073
	0.76547399	0.151705	0.01709	0.065731
	0.78139493	0.142539	0.014802	0.061265
	0.70038408	0.145858	0.031474	0.122284
	0.77246839	0.149055	0.015978	0.062499
	0.1393721	0.64977	0.163741	0.047117
Posture II	0.08333261	0.734705	0.157556	0.024406
	0.1262304	0.672221	0.162721	0.038828
	0.10477842	0.723614	0.14407	0.027537
	0.095901	0.713628	0.161922	0.028549
	0.12375778	0.748116	0.104327	0.0238
	0.12906179	0.738103	0.106699	0.026137
	0.13526255	0.76198	0.082374	0.020384
	0.09751504	0.649039	0.21497	0.038476
	0.18205851	0.683185	0.100583	0.034174
	0.09460856	0.706109	0.16927	0.030013
	0.10486092	0.758956	0.113677	0.022505
	0.1032312	0.713232	0.153851	0.029686
	0.10483996	0.76099	0.111962	0.022209
	0.09360699	0.7169	0.161521	0.027972
	0.13625302	0.765525	0.078912	0.019309
	0.1224168	0.720068	0.128504	0.029012
	0.10195895	0.724556	0.146286	0.027199

	0.07167455	0.679257	0.220184	0.028885
	0.12327556	0.682112	0.158435	0.036178
Posture Ⅲ	0.00052587	0.056164	0.940511	0.002799
	0.00042398	0.057704	0.939897	0.001975
	0.00062325	0.062329	0.934095	0.002952
	0.00050391	0.063394	0.933926	0.002177
	0.00035339	0.054148	0.943801	0.001698
	0.00043804	0.057645	0.939853	0.002064
	0.00034972	0.05664	0.941434	0.001577
	0.00059519	0.061482	0.935098	0.002825
	0.000373	0.055537	0.94233	0.001761
	0.0003465	0.053253	0.944708	0.001692
	0.00029652	0.051071	0.947171	0.001461
	0.00038438	0.056833	0.941008	0.001775
	0.00044733	0.05735	0.940067	0.002135
	0.00033562	0.051252	0.946704	0.001709
	0.0004369	0.056503	0.940948	0.002113
	0.00045015	0.058391	0.939058	0.002101
	0.00041804	0.056258	0.941317	0.002007
	0.00063406	0.065089	0.931449	0.002828
	0.00042074	0.056399	0.941164	0.002017
	0.00041999	0.057497	0.940122	0.001961
Posture IV	0.03302476	0.000515	0.006089	0.960372
	0.03317871	0.000541	0.006398	0.959883
	0.03342261	0.000529	0.006208	0.95984
	0.0328283	0.000498	0.005907	0.960767
	0.03543849	0.0009	0.010157	0.953505
	0.03703504	0.000644	0.006987	0.955334
	0.03167076	0.000395	0.004886	0.963049
	0.03133676	0.000419	0.005165	0.963079
	0.04224614	0.001787	0.017659	0.938308
	0.03466422	0.00047	0.005451	0.959415
	0.03572068	0.000647	0.007255	0.956377
	0.03127177	0.000498	0.006178	0.962052
	0.03403873	0.000368	0.004541	0.961053
	0.02969651	0.000306	0.004083	0.965914
	0.03531414	0.000721	0.008164	0.955802
	0.03558398	0.000536	0.006029	0.957851
	0.03407545	0.000578	0.006709	0.958637
	0.03024058	0.000421	0.005305	0.964034
	0.03332923	0.00052	0.006104	0.960047
	0.03222248	0.000561	0.0068	0.960417
