# Supplementary figures and images for: Stretchable and anti-impact iontronic pressure sensor with an ultrabroad linear range for biophysical monitoring and deep learning-aided knee rehabilitation
Source: Microsyst Nanoeng. 2021 Nov 17;7:92. doi: 10.1038/s41378-021-00318-2 (PMC8599697; doi:10.1038/s41378-021-00318-2)

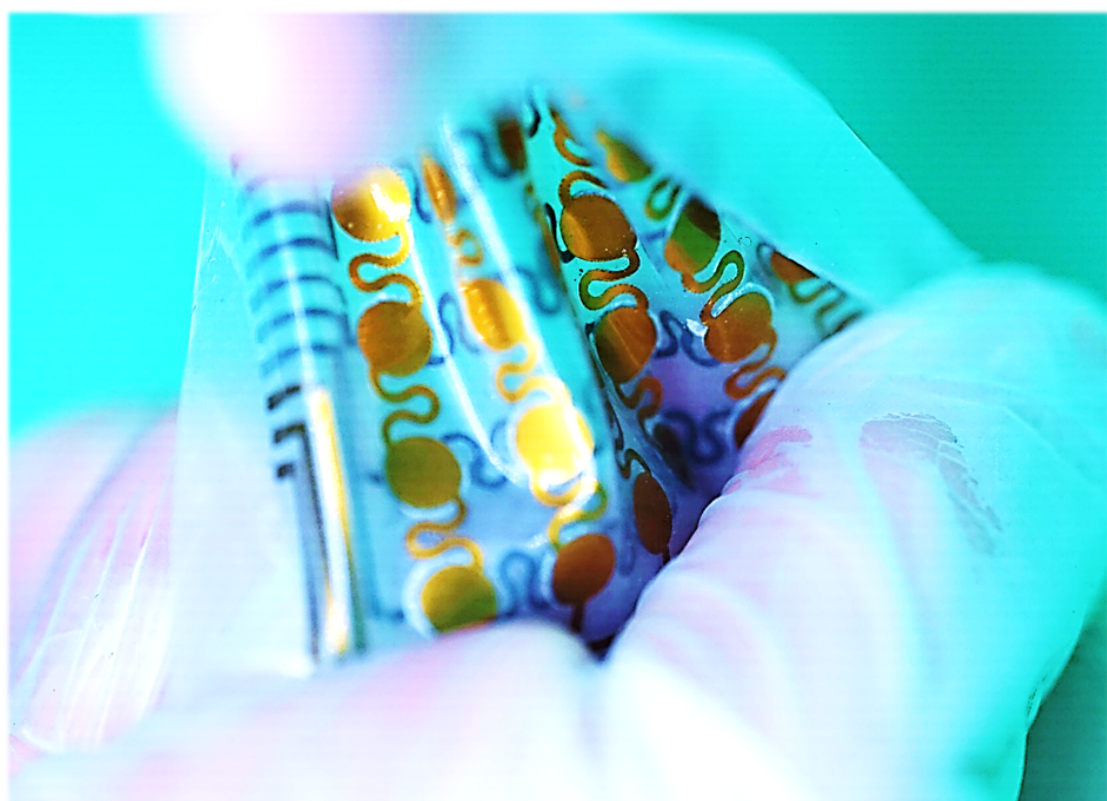

Supplement: Supplementary file 8 — Graphical Abstract [file 41378_2021_318_MOESM8_ESM.pdf]
